# PTSD in ICD-10 and proposed ICD-11 in elderly with childhood trauma: prevalence, factor structure, and symptom profiles

**DOI:** 10.3402/ejpt.v7.29700

**Published:** 2016-01-21

**Authors:** Tobias M. Glück, Matthias Knefel, Ulrich S. Tran, Brigitte Lueger-Schuster

**Affiliations:** Faculty of Psychology, University of Vienna, Vienna, Austria

**Keywords:** Latent class analysis, childhood abuse, World War II, institutional abuse, long term consequences, trauma severity

## Abstract

**Background:**

The proposal for ICD-11 postulates major changes for posttraumatic stress disorder (PTSD) diagnosis, which needs investigation in different samples.

**Aims:**

To investigate differences of PTSD prevalence and diagnostic agreement between ICD-10 and ICD-11, factor structure of proposed ICD-11 PTSD, and diagnostic value of PTSD symptom severity classes.

**Method:**

Confirmatory factor analysis and latent profile analysis were used on data of elderly survivors of childhood trauma (>60 years, *N*=399).

**Results:**

PTSD rates differed significantly between ICD-10 (15.0%) and ICD-11 (10.3%, *z*=2.02, *p=*0.04). Unlike previous research, a one-factor solution of ICD-11 PTSD had the best fit in this sample. High symptom profiles were associated with PTSD in ICD-11.

**Conclusions:**

ICD-11 concentrates on PTSD's core symptoms and furthers clinical utility. Questions remain regarding the tendency of ICD-11 to diagnose mainly cases with severe symptoms and the influence of trauma type and participant age on the factor structure.

The proposed ICD-11 classification for posttraumatic stress disorder (PTSD) aims to increase diagnostic specificity, practical reliability, and clinical utility (Maercker et al., 2013; Reed, 2010). Six core symptoms are evenly distributed on three symptom clusters (3×2): re-experiencing, avoidance, and hyperarousal. For a diagnosis, at least one symptom is required in each cluster. For elderly adults with a history of childhood trauma, it remains unclear whether (1) the proposed ICD-11 PTSD criteria and (2) the underlying factor structure also apply, and (3) how symptom severity influences classification. From a developmental perspective, childhood trauma is associated with more PTSD symptoms and greater impairment in older age compared with trauma in adult age (Ogle, Rubin, & Siegler, 2013). It was also reported that symptom configuration may differ in elderly persons (Averill & Beck, 2000). Only few studies investigated the influence of different trauma types on DSM-IV PTSD (American Psychiatric Association, 2000) symptom profiles: accidents, sexual abuse, and sudden death in student samples (Kelley, Weathers, McDevitt-Murphy, Eakin, & Flood, 2009; Lancaster, Melka, Rodriguez, & Bryant, 2014) and war- and crime-related trauma (Naifeh et al., 2008). No studies investigated these patterns in the elderly or used ICD criteria. Studies also did not use probabilistic methods, such as latent profile analysis (LPA) that increases validity (Goodyer, 2012), and models symptom severity as a latent variable.

With this study, we contribute to the ongoing discussion of the proposed ICD-11 PTSD classification by investigating the PTSD symptom configuration in two samples of now elderly survivors of childhood trauma (war-related vs. institutional childhood abuse). We replicate and extend previous studies on differences of PTSD prevalence and diagnostic agreement between ICD-10 (World Health Organization, 1992) and the ICD-11 proposal (Knefel & Lueger-Schuster, 2013; O'Donnell et al., 2014), retest the factor structure of ICD-11 PTSD (Forbes et al., 2015; Tay, Rees, Chen, Kareth, & Silove, 2015) in elderly persons, and explore latent symptom profiles of PTSD (Cloitre, Garvert, Brewin, Bryant, & Maercker, 2013).

## Method

### Participants

We combined samples from three research projects into one data set (*N*=399). For the sample of survivors of institutional abuse (IA), data from two studies on IA in institutions of the Catholic church in Austria and IA in institutions in Lower Austria (*n*=83) (Knefel & Lueger-Schuster, 2013; Lueger-Schuster et al., 2013) were included. For the sample of survivors of war-related childhood trauma, data from a study with now elderly survivors of World War II (WWII; *n*=316) were used (Glück, Tran, & Lueger-Schuster, 2012). For this study, only participants aged 60 years or older at the time of testing were included in the analysis. All studies were conducted according to the ethical regulations for clinical research in Austria. The institutional review board of the University of Vienna Studies allocated reference numbers for studies with survivors of IA (00011 and 00071). All participants provided written informed consent.

In total, 182 men (45.6%) and 217 women (54.4%) participated in the study; the WWII study sample was predominantly female, whereas the IA study sample was predominantly male (female: 62.3% vs. 24.1%, respectively; Pearson *χ*^2^(1)=38.76, *p<*0.001). Age ranged from 60 to 99 years (*M*=78.6 years, *SD*=9.2); participants of the WWII sample were older than participants of the IA sample (*M*=81.9 years, *SD*=6.84 vs. *M*=66.01, *SD*=5.72, resp.; *t*(397)=19.44, *p<*0.001).

### Measures

#### Posttraumatic Stress Disorder Checklist-Civilian Version

**Fig. 1:**
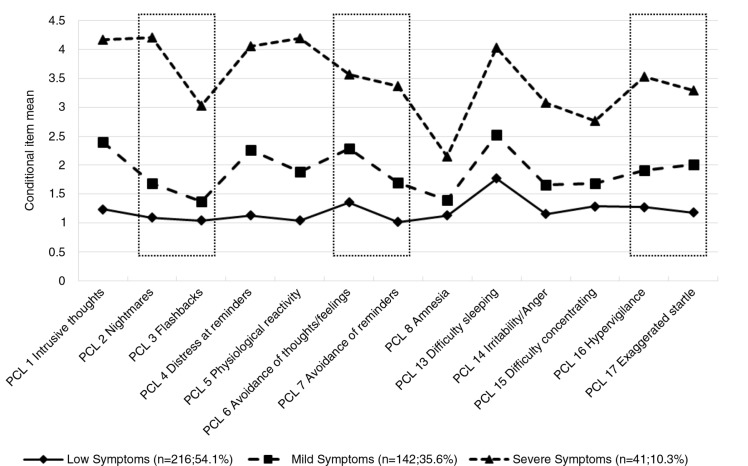
Conditional symptom severity means by cluster. Symptoms in boxes are proposed ICD-11 PTSD symptoms. PCL=Posttraumatic Stress Disorder Checklist.

The Posttraumatic Stress Disorder Checklist-Civilian Version (PCL-C) (Weathers, Litz, Herman, Huska, & Keane, 1991) assesses symptoms of PTSD in the past month according to DSM-IV criteria but can also be used to derive an ICD-10 and ICD-11 PTSD preliminary diagnosis. Seventeen items of the scale ask for five re-experiencing symptoms (criterion B), seven avoidance symptoms (criterion C), and five arousal symptoms (criterion D). These symptoms are rated on a 5-point scale from *none* (1) to *very* (5) and are considered symptomatic when indicated three or above. For this study, we only used items 1–8, and 13–17 as they are the symptoms included in the ICD-10 diagnosis of PTSD. For ICD-11 PTSD, items 2 and 3, 6 and 7, and 16 and 17 were used (see Fig. 1 for item contents). In this sample, algorithms for ICD-10 PTSD yielded a Cronbach's α=0.89, and for ICD-11 Cronbach's α=0.81.

#### Brief Symptom Inventory

The Brief Symptom Inventory (BSI) is a 53-item measure to assess current somatic and psychological symptoms and general distress (Franke & Derogatis, 2000). People rate different symptoms they experienced over the last 7 days on a 5-point rating scale (0*=not at all* to 4*=extremely*). Nine subscales and a Global Severity Index can be computed. The cut-off for clinically relevant symptoms is at the *T*-value of 63. For this study we only used the dimensions depression, anxiety, and somatisation. Psychometric properties are very good (Cronbach's α=0.96).

### Data analysis

First, we calculated proportions of individuals meeting the PTSD diagnosis according to ICD-10 and the ICD-11 proposal in both samples, as well as for all three criteria for both diagnostic systems. We used two-tailed binomial approximation *z* tests to compare proportions and Cohen's kappa to evaluate diagnostic agreement. We calculated proportions of clinically relevant comorbid depressive, anxiety, and somatic complaints for all PTSD groups. Secondly, we tested three-factor models of the ICD-11 PTSD structure (one-, two-, and three-factors) for goodness of fit. The one-factor model was a general factor model in which all items assessing the six ICD-11 PTSD symptoms were specified to load on a single factor. In the two-factor model, the items assessing symptoms of “re-experiencing” and “avoidance” were specified to load on one factor and the items assessing symptoms of “hyperarousal” were specified to load on the other factor (Forbes et al., 2015). Finally, we tested the proposed factor structure of PTSD in ICD-11 with a three-factor model, where symptoms of “re-experiencing”, “avoidance”, and “hyperarousal” presented one factor each. In the two- and three-factor models the latent factors were allowed to correlate.

We conducted all confirmatory factor analyses (CFAs) with Mplus (Muthén & Muthén, 2010). Since the symptom ratings are ordered categorical variables, parameters were estimated using the weighted least square mean- and variance-adjusted estimator (WLSMV), to provide robust parameter estimation, standard errors, and tests of model fit (Beauducel & Herzberg, 2006). The comparative fit index (CFI), the Tucker–Lewis index (TLI), and the root mean square error of approximation (RMSEA) were used to assess the model fit (CFI and TLI: good fit ≥0.95, acceptable fit ≥0.90; and RMSEA: good fit <0.06, acceptable fit <0.08) (Hu & Bentler, 1999). In order to compare the fit of the different models with the Bayesian Information Criterion (BIC), we reran the analyses using robust maximum likelihood estimation (MLR).

Thirdly, we determined the optimal number of latent classes in this sample based on the 13 ICD-10 PTSD symptoms. We applied LPA, which is based on a latent variable model that aims to find homogeneous groups of individuals in a given sample. We evaluated the model fit of a one-, two-, three-, and four-class solution using the BIC (based on the log-likelihood value), the likelihood ratio test statistic (*L*^2^), and the percentage of classification error. LPA was conducted using Latent GOLD (Vermunt & Magidson, 2005). In order to examine the possible relationship of age, gender, and sample, we used a multinomial regression model predicting class membership.

Finally, we combined results from the diagnostic algorithms with those from the CFAs and the LPA using cross tabulation to investigate to what extent the confirmatory and exploratory approaches matched the proposed ICD-11 diagnostic algorithm.

## Results

The PTSD prevalence in the total sample according to ICD-10 was significantly higher than according to the ICD-11 proposal (Pearson *χ*^2^(1)=189.38, *p<*0.001; Table 1); diagnostic agreement was 92.7% (κ=0.67, 95% CI 0.56 to 0.78); Significantly fewer individuals met the re-experience criterion according to ICD-11 than according to ICD-10. The avoidance symptoms do not differ between ICD-10 and the ICD-11 proposal, thus agreement was perfect here. While a similar proportion of individuals met the hyperarousal criterion, detailed analysis showed that about one-fourth of those who fulfilled the ICD-10 criterion failed to fulfill the ICD-11 criterion. Consequently, another fourth of those with ICD-11 hyperarousal did not fulfill this criterion in ICD-10.

**Table 1:** Prevalence and agreement of PTSD criteria according to ICD-10 and ICD-11 proposal, comorbidity, and agreement of classes with diagnostic status

								ICD-11 from ICD-10					
								
	ICD-10 PTSD		ICD-11 PTSD					Absent		Unchanged		Newly present	
								
Variable	*n*	%	*n*	%	*z*	*p*	*κ*	*n*	%	*n*	%	*n*	%
Full PTSD	60	15.0	41	10.3	2.02	0.043	0.67	24	6.0	370	92.7	5	1.3
Re-experience criterion	168	42.1	82	20.6	6.56	<0.001	0.53	86	21.6	313	78.4	0	0.0
Avoidance criterion	125	31.3	125	31.3	–	–	1.0	–	–	–	–	–	–
Hyperarousal criterion^a^	121	30.3	122	30.6	−0.08	0.936	0.64	30	7.5	338	84.7	31	7.8
BSI GSI T≥63^b^	41	70.7	32	78.0	−0.82	0.412		10	45.5	68	19.0	1	20.0
Depression	31	51.7	25	61.0	−0.92	0.358		8	33.3	56	15.1	2	40.0
Anxiety	36	60.0	32	78.0	−1.90	0.057		6	25.0	62	16.8	2	40.0
Somatisation	42	70.0	32	78.0	−0.90	0.368		12	50.0	122	33.0	2	40.0
Class													
Low Symptoms	0	0.0	0	0.0	–	–		0	0.0	216	58.4	0	0.0
Mild Symptoms	26	43.3	7	17.1	2.76	0.006		21	87.5	119	32.2	2	40.0
Severe Symptoms	34	56.7	34	82.9	−2.76	0.006		3	12.5	35	9.5	3	60.0

ICD-10, International Classification of Diseases—10th revision; ICD-11, International Classification of Diseases—11th revision; PTSD, posttraumatic stress disorder; BSI GSI, Brief Symptom Inventory: Global Severity Index.

a Avoidance symptoms do not change from ICD-10 to ICD-11 proposal, thus the same individuals met this criterion in both systems.

b *n*=384, full data unavailable for 15 participants.

The prevalence of PTSD varied across the samples; IA survivors had significantly higher rates of PTSD compared to WWII survivors according to ICD-10 (49.4% vs. 6.0%; Pearson *χ*^2^(1)=96.84, *p<*0.001) and according to the ICD-11 proposal (39.8% vs. 2.5%; Pearson *χ*^2^(1)=98.81, *p<*0.001).

Rates of comorbid conditions (clinical relevant symptoms in the domains of depression, anxiety, and somatisation) were consistently higher for PTSD according to the ICD-11 proposal than according to ICD-10, albeit not significant (Table 1).

All three specified confirmatory factor models demonstrated good fits to the data (Table 2). The fit of the one factor model was not improved substantially in the larger factor models. The obtained BICs, using MLR estimation, showed superior fit of the one-factor model over the two other models. Thus, our data did not support the two-factor model proposed by Forbes et al. (2015). On that account and with regards to parsimony, we adapted the analysis strategy and did not include the proposed two-factor structure of ICD-11 PTSD in further analyses. To rule out possible group effects, we repeated the CFAs for both samples (WWII vs. IA). For both samples analysed independently, the one-factor model also showed superior fit.

**Table 2:** Fit indices for ICD-11 PTSD factor models and fit of latent class models for ICD-10 PTSD symptoms

CFA	Model	χ^2^ (*df*)	CFI	TLI	RMSEA [95%-CI]	BIC^a^
	1-factor	18.678 (9)*	0.991	0.985	0.052 [0.016, 0.086]	6671.309
	2-factor	16.135 (8)*	0.992	0.986	0.051 [0.010, 0.087]	6673.889
	3-factor	6.875 (6)	0.999	0.998	0.019 [0.000, 0.070]	6677.937
LPA	Model	BIC	*L*^2^	*df*	*p*	Classification error, %

	1-class	11111.58	6600.16	52	<0.001	0.00
	2-class	10089.06	5493.79	66	<0.001	2.59
	3-class	9882.44	5203.33	80	<0.001	6.92
	3-class with correlated residuals^b^	9867.85	5182.75	81	<0.001	6.98
	4-class	9856.32	5093.36	94	<0.001	10.05

CFA, confirmatory factor analysis, using WLSMV estimation; CFI, comparative fit index; TLI, Tucker–Lewis index; RMSEA, root mean square error of approximation; 95%-CI, 95% confidence interval; BIC, Bayes information criterion; LPA, latent profile analysis; L^2^, likelihood ratio test statistic.

a Based on robust ML estimation.

b Model allowed correlated residuals for items 14 (irritability/anger) and 17 (exaggerated startle response).

* *p*<0.05.

In a third step, we aimed to identify latent groups of individuals with specific symptom profiles using LPA. We estimated a one-, two-, three-, and four-class model (Table 2). We estimated also a three-class model that allowed correlated residuals for items 14 and 17 (see Fig. 1 for item contents). The four-class model had the lowest BIC value. However, the classification error was considerably lower in the three-class model with correlated residuals and the additional fourth class did not provide further information. Therefore, the three-class model with correlated residuals was selected. Mean posterior assignment probabilities for all three classes were high, indicating high classification certainty (Class 1: 92.5%; Class 2: 93.0%; Class 3: 96.2%).

Based on the symptom severity profiles of the three classes, we assigned descriptive labels to each class. Class 1 was labelled “Low Symptoms”, Class 2 was labelled “Mild Symptoms”, and Class 3 was labelled “Severe Symptoms”. Mean symptom severity levels for each class are shown in Fig. 1. Multinomial regression analysis predicting class membership using gender, age, and study group (Table 3) had a significant fit on the data (*χ*^2^(6)=175.54, *p<*0.001) and explained 42.1% of the variance (Nagelkerke *R*^2^=0.421). Female gender was associated with the Mild Symptoms class rather than the Low Symptoms class. IA survivors were more likely to be in the classes with more severe symptoms than WWII survivors throughout all combinations.

**Table 3:** Multinomial regression model predicting class membership using gender, age, and study group

	Mild vs. Low Symptoms	Severe vs. Low Symptoms	Severe vs. Mild Symptoms
Age	1.01 [0.98–1.04]	0.95 [0.88–1.02]	0.94 [0.87–1.01]
Gender (female vs. male)	1.70 [1.04–2.76]*	1.25 [0.44–3.57]	0.74 [0.28–1.96]
Study group (IA vs. WWII)	15.38 [5.59–41.67]***	250.00 [38.46–>200]***	18.52 [2.98–111.11]**

Numbers are odds ratios (ORs) with 95% confidence intervals.

* *p<*0.05

** *p*<0.01

*** *p<*0.001.

Finally, we combined the results from the LPA and the diagnostic algorithms (Table 1). None of those who met either ICD-10 or ICD-11 PTSD were assigned to the Low Symptoms class. A significantly higher proportion of individuals with ICD-10 PTSD was assigned to the Mild Symptoms class, whereas a significantly higher proportion of individuals with ICD-11 PTSD was assigned to the Severe Symptoms class.

## Discussion

### Main findings

In this study, we investigated three different perspectives regarding the classification of PTSD in ICD. First, we found that the prevalence of PTSD decreased significantly from ICD-10 to ICD-11; however, the decrease in the re-experiencing criterion mostly explains this change. Secondly, in our sample a one-factor model of PTSD had better data fit compared to previously proposed two- or three-factor models. Nonetheless, two- and three-factor models showed also very good fit. Thirdly, three latent severity groups of individuals emerged when we analysed symptom profiles in the whole sample. Symptom severity group membership was a good predictor for PTSD diagnosis and type of trauma.

### PTSD in ICD-10 and ICD-11

Significantly fewer individuals met PTSD criteria in ICD-11 than in ICD-10. Very similar to our results, other studies found higher prevalence rates of PTSD in ICD-10 vs. ICD-11 in different samples (9.0% vs. 3.3% in adult injury patients O'Donnell et al., 2014, 13.0% vs. 6.0% in West-Papuan refugees Tay et al., 2015, and 4.4% vs. 3.2% in the general population Stein et al., 2014). The prevalence decrease in our study is mainly attributable to the stricter definition of the re-experiencing criterion in ICD-11: while 42.1% met this criterion in ICD-10, only 20.6% met it in ICD-11. Individuals who fulfilled the ICD-11 re-experiencing criterion were a complete subgroup of those fulfilling the re-experiencing criterion in ICD-10. This is in line with previous research (O'Donnell et al., 2014; Tay et al., 2015) and the pattern seems to be unspecific for type of trauma and culture. ICD-11 proposes to restrict criteria only to the core-elements specific for PTSD (Maercker et al., 2013). This entails that the re-experiencing criterion includes only reliving the trauma in form of nightmares or flashbacks, accompanied by fear and horror (Brewin, 2013). It is controversial whether this approach is valid as 21.5% of our sample suffer from clinically relevant intrusive symptoms but do not fulfill the ICD-11 re-experiencing criterion. The exclusion of other re-experiencing related symptoms, such as intrusive images without dissociative character, may not adequately capture the phenomenology and the needs of individuals suffering from symptoms following exposure to a traumatic event (Bisson, 2013). The DSM-5 definition of re-experiencing in PTSD (American Psychiatric Association, 2013) did not follow this approach (Friedman, 2013) and remains similar to DSM-IV and ICD-10. For the re-experiencing criterion to be fulfilled, DSM-5 requires one symptom out of five symptoms that cover various forms of re-experiencing symptoms focussing on the intrusive nature in contrast to ruminative processes. Consequently, individuals who suffer from symptoms of re-experiencing not defined as core symptoms of ICD-11 PTSD need to be studied with regard to comorbidity, treatment needs, and treatment responsiveness.

The hyperarousal criterion also differs from ICD-10 to ICD-11 and we found partly overlapping subgroups. The ICD-11 working group aimed to focus on trauma-specific types of hyperarousal (i.e., hypervigilance and exaggerated startle response) and excluded more unspecific types of hyperarousal (e.g., difficulties with sleep or concentration) (Maercker et al., 2013). Sleeping difficulties, for example, are highly prevalent in the general population and increase with older age (Ohayon, Carskadon, Guilleminault, & Vitiello, 2004). In elderly samples this may likely bias PTSD prevalence rates.

### Factor structure of PTSD in ICD-11

All factor models tested in the current sample had a good fit on the data. A one-factor model was superior compared to the two-factor model proposed by Forbes et al. (2015) and compared to the three-factor model proposed by the ICD-11 working group (Maercker et al., 2013). A one-factor solution appears unusual for PTSD, regardless of the classification system used—ICD (Forbes et al., 2015) or DSM (Yufik & Simms, 2010). Various reasons might explain the difference between our results and the results of Forbes et al. (2015): (1) the assessment of the relevant symptoms differed between the two studies. While we used a self-report measure for PTSD with one item per symptom, Forbes et al. used the CAPS (Blake et al., 1995) that includes two items (frequency and intensity) for each symptom. (2) Results may depend on trauma type or age at traumatisation. We studied elderly persons with partially severe and long-lasting experiences of childhood trauma. Forbes et al. investigated PTSD symptoms in injury survivors with an average age of 40 years. Symptom presentation after prolonged trauma in childhood might differ from that after traumatic injury in adulthood which may influence the factor structure (Yufik & Simms, 2010). (3) Time since trauma may be an additional influencing factor (Yufik & Simms, 2010). While participants in our study were exposed to traumatic events that took place decades ago, Forbes et al. analysed data from a 6 year follow-up study. (4) Age at assessment may also affect symptom presentation. These aspects call for further investigation, testing for the invariance of the ICD-11 PTSD factor model across various age groups and groups of persons with different types of trauma.

### Symptom severity, comorbidity, and type of trauma

No person with a PTSD diagnosis (ICD-10 or ICD-11) was allocated to the Low Symptoms class. PTSD diagnosis was associated with class membership differentially for ICD-10 and ICD-11: persons who met criteria for PTSD in ICD-10 had almost equal chances to be allocated to the Mild and Severe Symptoms class. In contrast, persons who met criteria for ICD-11 PTSD were much more likely to be allocated to the Severe Symptoms class. In our sample, ICD-11 appears thus more specific to persons with severe symptoms of PTSD and excludes those with milder symptoms. Persons suffering from less-than-severe trauma-related distress may thus remain undiagnosed according to ICD-11 PTSD criteria.

We did not find a decrease in comorbid conditions in our sample with ICD-11 compared with ICD-10. This was unexpected, because the focus on core-elements of PTSD in ICD-11 aimed to reduce comorbidity with other mental disorders, and this was a major critique of ICD-10 (Maercker et al., 2013). In our sample, ICD-11 excluded individuals with milder PTSD symptoms from a PTSD diagnosis, which was not the case for ICD-10. The individuals with severe symptoms were also those who displayed the most comorbid conditions in ICD-10. Thus, ICD-11 diagnosed specifically individuals with severe symptoms and high comorbidity rates with PTSD (Table 1).

The strongest predictor for symptom severity class and PTSD was type of trauma, which in our study is mostly equivalent to the study group (IA vs. WWII). The Severe Symptoms class included mainly IA survivors, and the experience of complex childhood trauma is a major risk factor for PTSD (Briere, Kaltman, & Green, 2008; Cloitre et al., 2009). Although war-related experiences are similarly considered complex and adverse, especially when they happen in younger age (Bramsen & Van der Ploeg, 1999), it seems that in our sample long-lasting effects on current mental health have either faded over the lifespan, were buffered by unknown factors, or have not been as detrimental in comparison to the effects of IA (Böttche, Kuwert, & Knaevelsrud, 2012). There was a partial gender effect: women were more likely in the Mild than in the Low Symptoms class; however, no gender effects were present for the Severe Symptoms class. This might be a result of the distribution of study groups to the severity classes. Current age had no effect on the allocation of individuals to symptom severity classes. Future research needs to elucidate the factors that are mainly responsible for high symptom distress for these trauma types and whether there is a critical threshold of trauma complexity which makes remission over the life span unlikely.

### PTSD in older age

As noted earlier, childhood trauma is associated with greater impairment in old age compared to traumatic events experienced in other developmental stages such as, for example, adulthood (Ogle et al., 2013). However, it has also been reported that elderly who report early-life traumatisation present a decline in PTSD symptom severity over the life course (Böttche et al., 2012) and that the symptom configuration may differ in elderly persons (Averill & Beck, 2000). With the possibility of a symptom change over the lifespan, posttraumatic symptoms could also change from “typical” PTSD symptoms to somatoform disorders or other trauma-related mental health problems (Freitag, Braehler, Schmidt, & Glaesmer, 2013; Noll-Hussong et al., 2012) and also more somatic health problems may be present in older age (Glaesmer, Brähler, Gundel, & Riedel-Heller, 2011; Pietrzak, Goldstein, Southwick, & Grant, 2012). Therefore, a differentiated analysis of symptom profiles seems more informative than the investigation of overall symptom severity (Yehuda et al., 2009).

With regard to these aspects, we found that persons with severe symptoms not only had a higher likelihood of being diagnosed with PTSD but also showed the highest amount of comorbid mental health problems such as depressive, somatisation, or anxiety symptoms. This was especially the case for survivors of complex and severe childhood trauma such as IA. All these problems add to the general symptom burden that affects the current health of survivors. However, with the data used in this study, we were not able to investigate whether there were particularities in the symptom representation in our samples compared to other age groups. In general, results regarding the course and severity of PTSD symptoms over the lifespan are still inconclusive, which questions whether current PTSD classification is adequate to describe symptom patterns in older age (cf. Böttche et al., 2012). These issues need to be addressed in future studies that compare posttraumatic reactions in different age groups of the sample population, that is, who have experienced comparable traumatic events.

### Limitations

The generalisability of the current findings is limited by the following aspects: (1) the data were combined from studies that assessed different and specific types of childhood trauma (WWII-related and IA) and current posttraumatic symptoms with a cross-sectional design. Although it was a strength and an aim of the current study to compare different types of trauma in different samples, we cannot rule out that our results were confounded by variables that could not be controlled for. (2) We used self-report questionnaires and not clinician-administered interviews. The questionnaires were originally designed to assess DSM-IV PTSD symptoms because a validated measure for ICD-11 PTSD symptoms is still lacking. Furthermore, the ICD-11 proposal of PTSD includes functional impairment, which was not assessed with our measures. (3) The reporting of traumatic events may be biased by recall problems in the elderly. Future investigations on the structure and symptom configuration of ICD-11 PTSD should be performed in large samples assessing different trauma types, thus accounting for potential biases to all over prevalence. Furthermore, as soon as there is a final agreement on the ICD-11 core diagnostic features of PTSD, studies need to replicate findings using validated measures designed for ICD-11.

### Implications

This study addresses important issues regarding the diagnostic and clinical utility of the proposed ICD-11 criteria of PTSD, to be published in 2017. As expected, the prevalence of PTSD decreases significantly with the ICD-11 proposal as it aims to assess only PTSD core symptoms and to lower the inflation bias by comorbid symptoms that are better explained by other mental disorders. However, comparing ICD-10 and ICD-11, we did not find a specific profile of trauma-related symptoms but rather a grading of severity classes. ICD-11 PTSD seems much more specific to persons with severe trauma symptoms. Rates of comorbid conditions did not change from ICD-10 to ICD-11 in our sample, failing to support the aim of ICD-11 to reduce comorbidity of PTSD with other mental disorders. In our study, a one-factor model of PTSD showed the best fit, which is in contrast to previous research (Forbes et al., 2015; Yufik & Simms, 2010). This result calls proposed factor structures into question, at least for elderly persons with a history of complex trauma. With a change of PTSD criteria in classification systems, individuals who suffer from less-than-severe symptoms might lose their access to mental health services. More investigations are needed regarding key issues such as symptom configuration, comorbidity, factor structure, and trauma specificity to support and consolidate the proposed criteria for PTSD in ICD-11.
